# Recent insights into Nanoemulsions: Their preparation, properties and applications

**DOI:** 10.1016/j.fochx.2023.100684

**Published:** 2023-04-20

**Authors:** Abeeda Mushtaq, Sajad Mohd Wani, A.R. Malik, Amir Gull, Seema Ramniwas, Gulzar Ahmad Nayik, Sezai Ercisli, Romina Alina Marc, Riaz Ullah, Ahmed Bari

**Affiliations:** a Division of Food Science and Technology, Sher-e- Kashmir University of Agricultural Sciences and Technology-Kashmir, Srinagar, Jammu and Kashmir, India; b Division of Fruit Science, Sher-e-Kashmir University of Agricultural Sciences and Technology of Kashmir, Srinagar, Jammu and Kashmir, India; c Department of Food Science and Technology, University of Kashmir, Srinagar, Jammu and Kashmir, India; d University Centre for Research and Development, Chandigarh University, Gharuan, Mohali 140413, Punjab, India; e Department of Food Science and Technology, Government Degree College Shopian, J&K, India; f Department of Horticulture, Faculty of Agriculture, Ataturk University, 25240 Erzurum, Turkey; g Food Engineering Department, Faculty of Food Science and Technology, University of Agricultural Sciences and Veterinary Medicine, 400372 Cluj-Napoca, Romania; h Department of Pharmacognosy, College of Pharmacy, King Saud University, Riyadh, Saudi Arabia; i Department of Pharmaceutical Chemistry, College of Pharmacy, King Saud University, Riyadh, Saudi Arabia

**Keywords:** Nanoemulsions, Emulsions, Shelf-life, Vegetables, Fruits, Preservation

## Abstract

The ever-increasing demand for healthy diet by consumers has prompted the research adopting cutting-edge methods that can maintain the quality of fruits and vegetables without the use of preservatives. Emulsion based coating approach has been regarded as a viable way to extend the shelf life of fresh produce. New opportunities are being created in a number of industries, (medicines, cosmetics and food) because of new advancements in the developing field of nanoemulsions. Nanoemulsion based methods are efficient for encapsulating the active ingredients including antioxidants, lipids, vitamins and antimicrobial agents owing to the small droplet size, stability and improved biological activity. This review provides an overview of recent developments in preserving the quality and safety of fresh-cut fruits & vegetables with nanoemulsion as a carrier of functional compounds (antimicrobial agents, antibrowning/antioxidants and texture enhancers). In addition, material and methods used for fabrication of the nanoemulsion is also described in this review. In addition, material and methods used for fabrication, of the nanoemulsion is also present.

## Highlights

• In the agro-food related industries, nanoemulsions have been regarded as an efficient and most suitable form of technology.
• An effective method for creating innovative products is the creation of food-grade nanoemulsions.
• Vegetables, fruits, beverages, food packaging, and the dairy industries might all use edible nanoemulsions can meet the market's large-scale demand.
• The findings of current research indicate the potential advantages of utilising natural nanoemulsion rather than traditional emulsion.

## Introduction

An emulsion is a colloidal system, lyophobic in nature consisting of two immiscible liquids (usually oil and water); first liquid is homogeneously dispersed in second liquid having the shape of circular globules. The liquid surrounding the continuous phase constitutes droplets that are small sized ranging from a diameter of 0.01 to 100 µm (Tan and McClements, 2021). Depending on the stability and droplet size, emulsions are categorized as nanoemulsions, microemulsions and macroemulsions or coarse emulsions (Jose et al., 2022).

Nanoemulsions also known as submicron emulsions, ultrafine emulsions and mini-emulsions, are heterogeneous colloidal particulate system comprising of a minimum of two immiscible liquids, one is dispersed into another as tiny drops in the range of 20 to 500 nm, with size measuring from 50 to 200 nm is transparent and till 500 nm appears milky. Out of the two immiscible liquids one must be aqueous in character and another oleaginous (Lago et al., 2019). Nanoemulsions can be optically clear or somewhat murky depending on the size of the droplets. The opacity represented as turbidity (τ) is determined by measurement of transmission. The size of droplets besides determining the optical property and stability also influences the release behavior and rheology. Therefore nanoemulsions are more appropriate than micro-emulsions for different applications. The miniature size of particles in nanoemulsions has many important consequences over other forms. Nanoemulsions help in maintaining the stability and increasing physicochemical properties (Nasr et al., 2022). Nanoemulsions impart aqueous form to drugs making them water insoluble and increasing the bioavailability due to rapid digestion. Play a crucial part in removing the absorption variability and also contributes in protection from oxidation and hydrolysis as oil-in-water nanoemulsion avoids attack by air as well as water. The effectiveness of a drug can be increased by using nanoemulsion for distribution, which lowers the overall dose and side effects (Garcia et al., 2022). Nanoemulsion comprise of an aqueous phase, oil phase and emulsifier. Water is mostly used to obtain the aqueous phase. Other polar compounds used include carbohydrates, proteins, co-solvents (polyols and simple alcohols), acids, bases and minerals. Various non-polar components used for preparing the oil phase formulation are essential oils, diacylglycerols, triacylglycerols, monoacylglycerols, fat substitutes, mineral oils, free fatty acids, weighting agents, waxes, lipophilic compounds and vitamins (Pavoni et al., 2020). Non-polar mineral oils, essential oils, waxes, lipid substitutes, oil-soluble vitamins, weighting agents and many lipophilic compounds can also act as oil phase. The most ideal nanoemulsion formulations are long-chain triacylglycerols due to their availability, economical, nutritional and functional factors. Polar solvent and a cosolvent form the single aqueous phase of a nanoemulsion. The stability properties and formation of nanoemulsions mostly depends on the oil phase physicochemical parameters, such as its interfacial tension, refractive index, viscosity, polarity, chemical stability, density, water-solubility and phase behavior (Jafari et al., 2013).

Generally, two immiscible liquids used commercially namely water and oil; give nanoemulsions of water-in-oil (W/O) type or oil-in-water (O/W) type. W/O nanoemulsions contain small drops of water dispersed in an oil phase while O/W nanoemulsions constitute few oil drops dispersed in an aqueous phase (Jafari et al., 2017). The emulsifiers adopted for coating of droplets in W/O nanoemulsions are generally lipophylic or hydrophobic while as for O/W nanoemulsions hydrophilic emulsifier is used. O/W nanoemulsions are ideal for making edible coatings, as diverse lipophilic compounds having antimicrobial and antioxidant effects can be incorporated within a hydrophilic polymeric system (Zambrano-Zaragoza et al., 2018). Usually, O/W nanoemulsion is primarily fabricated by homogenization of a high-melting lipid and a hydrophilic surfactant present in an aqueous phase at a temperature higher than the melting point of lipids. Then cooling of the system is done lower than the melting point of the lipid phase, due to which crystals of oil droplets are formed (Assadpour et al., 2017).

Nanoemulsions can be made extremely gel like or viscous with a very low concentration of droplets than the conventional emulsions and can be consequently utilized to create compounds having new textural properties and low calories. Nanoemulsions having low fats face challenges, during preparation because of the low content of oil, they must be made using fillers/binders or emulsifiers to enhance their viscosity. Pickering emulsions have proved beneficial for preparing nanoemulsions containing highly stable low fats as the solid material used for stabilization, acting as a thickening agent (Prakash et al., 2018). The enhanced properties of nanoemulsions can find innovative, appropriate and novel industrial applications (Dasgupta et al., 2019). In this regard, the primary objective of this review is to provide updated information related to the usage of nanoemulsion as an advanced edible coating or as nanocarrier of antibrowning/antioxidants, texture enhancers and antimicrobials for fruits and vegetables.

## Nanoemulsion preparation

Selecting a suitable ingredient is important for the successful formulation of a nanoemulsion. Nowadays, safe or natural (GRAS: Generally Recognized As Safe) components are used for the formulation of nanoemulsions which include surfactants (Tweens, spans) and food biopolymers (proteins, polysaccharides) because of their low toxicity (Sridhar et al., 2021). Edible film and coatings are usually grouped based on their structural material used like lipids, proteins, polysaccharides, or composite. Commonly three methods are utilized for increasing the chemical stability of nanoemulsion that includes the incorporation of antioxidants or chelating agents, manipulation of interfacial characteristics (e.g. charge, thickness and chemical reactivity) and controlling the atmospheric conditions (temperature, oxygen levels, pH and light) (Liu et al., 2019). Different materials used for the preparation of nanoemulsions with their properties are shown in Table 1.

**Table 1:** Material used for the preparation of nanoemulsions with their properties.

**Material used**	**Properties**	**Examples**
Oil/lipids	Possess good barrier properties against moisture which help in preventing physiological loss of food stuff	carnauba wax, paraffin, waxes (beeswax), candelilla wax, rice bran wax), organic fatty acids, acetoglycerides and resins (wood rosin, shellac)
Proteins	Proteins are adsorbed at the water and oil interface, forming a viscoelastic coating which prevents droplets of emulsion from physical deterioration, coalescence in particular	Wheat gluten, casein, whey protein, collagen, gelatin, keratin, rice bran protein, soy protein, peanut protein, corn-zein cotton seed protein and pea protein
Polysaccharides	Form strong hydrogen bonds when dissolved in water and transmits good mechanical properties to edible coating and films	Gum arabic, pectins, modified starch, modified alginate, modified celluloses, and some cellulose or galactomannans derivatives, gums, alginates, carrageenans, starches, pectins
Surfactants	Reduces interfacial tension and prevents droplet aggregation and stabilizes nanoemulsions	Cremophor EL (Polyoxyl-35 castor oil), lecithin, sodium deoxycholate (bile salt), β-lactoglobulin, sodium dodecyl sulfate
Emulsifiers	lowers interfacial tension and inhibits aggregation of droplets	Phospholipids (dairy, soy or egg lecithin), small molecule surfactants (Spans, Tweens), amphiphilic polysaccharides (modified starch, gum Arabic), amphiphilic proteins (caseinate, WPI)
Weighting agents	Compounds which are incorporated in oil droplets so that their densities match with the continuous phase in surrounding	Dense lipophilic materials (BVO, Ester Gums, SAIB)
Texture modifiers	Compounds which gel the aqueous phase or improve the viscosity. These can also be used to offer desired textural features, or prevent gravitational separation	Polyols (sorbitol, glycerol), sugars (HFCS, sucrose), proteins (WPI, gelatin, SPI)and polysaccharides (carrageenan, xanthan, alginate, pectin)
Ripening inhibitors	Potential hydrophobic compounds which slow down or inhibit Ostwald ripening when added in oil drops	Lipophilic substances with very less water solubility (LCT, esters gums)
Plasticizers	decreases brittleness and cohesiveness, imparts flexibility and improves toughness and thermoplastic properties of the polymer	Monosaccharides, oligosaccharides (honey, glucose, sucrose, and fructose), polyols (sorbitol, polyethylene glycols, glycerol), lipids and its derivatives (fatty acids, phospholipids), ethylene glycol, glycerol, sucrose, sorbitol, xylitol.

### Oil or lipids

Resins (wood rosin, shellac), paraffins, waxes (beeswax), rice bran wax, carnauba wax, candelilla wax), organic fatty acids, neutral lipids and acetoglycerides can be utilized in edible film and coating formulation (Umaraw and Verma, 2017) attributed to their hydrophobic nature and barrier properties against moisture thus inhibiting physiological degradation of food products. Lipid-based nanoemulsions also enhance solubilization by increasing the absorption. Furthermore, various lipids impart a glossy appearance when applied on the food product. Lipids or resins used for making coatings and films have reduced mechanical properties because they are not polymers. Nanoemulsions usually consist of 5 to 20% lipid/oil droplets in (O/W) emulsion, but the percentage of lipid can be much higher (until 70%) in some cases (Singh et al., 2017). Due to reduced optical and mechanical properties, films based on lipids are moderately thick, usually brittle and nontransparent. Another drawback of lipid-based coatings is that they stick poorly to food commodities having hydrophilic surfaces.

### Proteins

Proteins exhibit amphiphilic properties due to the presence of both hydrophilic (polar) and hydrophobic (nonpolar) amino acid residues that are exposed on their surface. Studies have demonstrated that numerous edible proteins can serve as effective emulsifiers in (O/W) nanoemulsions by creating a protective layer around the oil droplets, which stabilizes them through a combination of steric hindrance and electrostatic repulsion (Costa et al., 2019). Despite their ability to stabilize emulsions, proteins tend to be sensitive to alterations in pH and ionic strength, as their electrical properties play a significant role. Specifically, they have a propensity to aggregate near their isoelectric points or at high salt concentrations, which results in a weakening of the electrostatic repulsion. (Ozogul et al., 2022). Proteins have attracted more interest due to their capabilities of forming variety of films and coatings. Natural sources used for protein-based edible coating fabrication includes whey protein, casein, gelatin, collagen, keratin, mung bean protein, rice bran protein, pea protein, soy protein, cottonseed, corn-zein protein, wheat gluten and peanut protein (Flores-López et al., 2016). In an aqueous solution, protein interactions create hydrophobic interactions, hydrogen bonds, or ionic bonds. The water solubility of proteins depends on their constituent functional groups. Edible coating and films generally exhibit small moisture barrier properties but good permeability to gases and mechanical properties when the proteins selected tend to form hydrogen or ionic bonds (Umaraw and Verma, 2017). Low water soluble proteins are generally less permeable to vapours and gases. Due to high mechanical, physical features and film forming capability, proteins fit in particular needs for different food products. However, proteins have a limited number of applications due to their comparatively high chance of causing allergenic reactions.

### Polysaccharide

Polysaccharide based edible coatings are generally utilized for fruits and vegetables owing to their proper flexibility and ability to adhere to the surface of the product. Amphiphilic structured polysaccharides such as modified celluloses, modified alginate, some galactomannans, modified starch, pectins and gum arabic or cellulose derivatives gums, carrageenans, alginates are likely used as emulsifiers (Versino et al., 2016). They have moderately low cost and are highly available. The majority of polysaccharides form strong hydrogen bonds when dissolved in water, transmits good mechanical effect to edible coating, and films formed. Polysaccharides are permeable to moisture due to their hydrophilic nature, which reduces their capability to avoid water loss from foodstuff. Generally, polysaccharides have a very low effect (or none at all) on flavour or appearance of food and do not cause any allergic reactions unlike proteins. The disadvantage of using polysaccharide for nanoemulsions is their high water vapour permeability and water solubility. They are undesirable for liquid items due to their low moisture barrier qualities as well (Kong et al., 2022).

### Surfactants

They are amphiphilic molecules that impart stability to the nanoemulsions by preventing droplet aggregation and reducing interfacial tension. Surfactants are readily adsorbed at the oil and water interface providing electrostatic, steric, or dual electro-steric stability. Lecithin (phosphatidylcholine) is the frequently used surfactant in the nanoemulsions, obtained from soybean or egg yolk. Surfactants like bile salts (sodium deoxycholate) and cremophor EL (Polyoxyl-35 castor oil) (Zhang et al., 2016) have been used in markets. Surfactant adsorption at an interface encapsulating the dispersion medium and droplets, which lowers interfacial tension, is a common method for stabilizing nanoemulsions.

Additional frequently used surfactants comprise those which belong to amphiphilic proteins like β-lactoglobulin (Ali et al., 2016), casein, poloxamer family, sodium dodecyl sulfate (Uluata et al., 2016), polysaccharides (e.g., starch derivatives, gums) (Combrinck et al., 2014) and polyethylene glycol (PEG) consisting of block copolymers. A variety of cosurfactants can be set in pairs with surfactants and the emulsification effectiveness (region area of nanoemulsion) would come to the selection perspective. The surfactants alone are not adequate to decrease surface tension; in such conditions, the reduction can be achieved by adding a cosurfactant to the formulation. Commonly used cosurfactants in nanoemulsions (food grade) are medium or short chain alcohols like cosolvents and ethanol like polyols like sorbitol, propylene, glycol and glycerol. Surfactants are ionic, non-ionic and zwitterionic. (Hasan et al., 2020).

#### Ionic surfactants

They can be anionic or cationic in nature. Normally edible anionic surfactants like citric acid esters of mono and diglycerides of fatty acids (CITREM), sodium lauryl sulfate, sodium dodecyl sulfate, sodium caseinate and diacetyl tartaric acid esters of mono and diglycerides (DATEM) provide more benefits in food manufacturing in comparison to cationic ones like β-lactoglobulin and lauric arginate. Ionic surfactants prevent droplet aggregation by electrostatic repulsive forces. The drawback of ionic surfactants is that at high concentrations they are capable of causing irritation thus restricting their usage in commodities requiring high levels of surfactant (McClements and Rao, 2011).

#### Non-ionic surfactants

They are an essential constituent in the preparation of edible nanoemulsions. The major advantages include the capability of forming stable, low toxic nanoemulsions which do not cause irritation. Nonionic surfactants decrease aggregation by hydration, thermal fluctuations and steric hindrances (Silva et al., 2015). Nonionic surfactants which are being extensively being used in the food industry include polyglycerol esters of fatty acids, sorbitan monooleate, polyoxyethylene ether surfactants (Brij 97), sucrose monopalmitate and ethoxylated sorbitan esters (Spans and Tweens) (Goindi et al., 2016).

#### Zwitterionic surfactants

These have two or more ionizable groups with opposing charges on the same molecule. As a result, these can possess a net neutral, negative or positive charge which depends on the pH of the solution. Commonly utilized zwitterionic surfactants are phospholipids having GRAS status thus allowing their utilization in food, like lecithin (McClements and Rao, 2011, Goindi et al., 2016). The main advantages include higher surface activity, high biodegradability, high foam stableness, low irritating and toxicity, lower critical micelle concentration (CMC) and higher water solubility. But the zwitterionic surfactants are costly compared to other class of surfactants so far (Sarkar et al., 2021).

### Stabilizers

Usually used to aid in the preparation and everlasting stability of nanoemulsions consisting of texture modifiers, emulsifiers, ripening inhibitors and weighting agents (McClements and Jafari, 2018). If an aqueous phase and an oil phase are combined during homogenization, a rapid breakdown of the system through different mechanisms, like gravitational separation, droplet flocculation, ostwald ripening and coalescence will occur. Consequently, it becomes essential to include a variety of stabilizers to improve the long lasting stability of nanoemulsion. The distribution of stabilizers on an element can make solid particulate, monolayer and multilayer nanoemulsions (Aswathanarayan and Vittal, 2019). Moreover, few surfactants have been detected as irritants, due to which they have limited applications in food. This led to the formulation of (W/O) emulsion devoid of surfactants. These nanoemulsions are free from surfactants and can be made by lowering the temperature of the continuous phase less than its melting point, due to which kinetically stable emulsion is formed which constitutes lyotropic liquid crystalline nanostructure (Chen et al., 2018).

### Emulsifiers

Surface active elements adsorbed at the exterior of freshly formulated oil droplets at the time of homogenization. Once they get adsorbed this facilitates further droplet disintegration by decreasing interfacial tension, thus lowering droplets size at the time of homogenization. Emulsifiers are mostly included for relieving the droplet disintegration by using top down approaches which decrease the droplet size and prevent aggregation thus maintaining stability for a long period (Dasgupta et al., 2017, Jain et al., 2018). The addition of emulsifier is compulsory in the nanoemulsions formulation. If emulsifiers are not incorporated, there will be a rapid breakdown of nanoemulsion due to increased surface area. The choice of an appropriate emulsifier and its concentration in the oil-phase depends on the requisite coverage of surface for acquiring stable systems as well as the principles of surface adsorption and reorganization at oil in water interfaces, to make a well-organized emulsification process (Donsì and Ferrari, 2016). The concentration of emulsifiers also has a vital role in the coverage and interfacial tension of droplet surface, which affects breakup effectiveness of droplet, adsorption kinetics and droplet coalescence. Emulsifiers improve the kinetic stability of an emulsion and lengthen the food's storage period. These emulsifiers significantly affect the texture, structure and functionality of many food products in addition to stabilising the emulsion (McClements and Jafari, 2018). Emulsifiers generally are of two types: (1) polymers, like peptides/proteins and poly (vinyl alcohol) and (2) tiny, amphiphilic molecules known as surfactants. The later could be charged, mostly zwitterionic or anionic compound, like oleates, phospholipids and lactic acid esters, or can be a nonionic compound, along with sorbitan derivatives, aliphatic alcohols, monoacylglycerols and their esters (Espitia et al., 2019).

### Texture modifiers

Materials that gel or thicken the aqueous phase. In commercial goods texture modifiers are frequently incorporated to enhance the emulsion stability by reducing movement of droplets. However, they can also give textural characteristics which are desirable like gel strength, thickness, richness, or creaminess (Liu et al., 2019). Commonly used texture modifiers in food industries are biopolymers which include polysaccharides (carrageenan, pectin, starch, guar gum, alginate, xanthan) or proteins (egg, vegetable proteins, milk). Hydrocolloids have been frequently employed in food compositions because of their ability to gel or thicken when added to aqueous liquids. They could be utilised in nanoemulsions to modify the texture of food or improve the stability of gravitational separation. Proteins also act as gelling and thickening agents (Primozic et al., 2017).

### Weighting agents

Hydrophobic additives having high densities are incorporated in nanoemulsions to minimize the separation of oil drops. Weighing agents are the materials with higher density that increase oil droplet density and match with the aqueous phase. Usually, creaming is prevented by weighing agents in nanoemulsions having a low density oil phase. Besides, these weighting agents increase the refractive index as well as the cloudiness of emulsion and decrease the turbidity of emulsion. Damar gum, protein, Ester gum and sucrose acetate isobutyrate (SAIB) are the commonly applied weighing agent used for escalating the oil phase density (Stounbjerg et al., 2018). Weighting agents reduce the driving force essential for the gravitational separation, resulting in the development of a stable emulsion. Some of the mainly utilized weighting agents in food and beverage industry consist of sucrose-acetate isobutyrate, brominated vegetable oil, damar gum and ester gum (McClements and Rao 2011).

### Ripening inhibitors

Extremely hydrophobic molecules having reduced water solubility are added into the dispersed phase of nanoemulsion to avoid expansion of droplets due to ostwald ripening, resulting in disturbing the effect of mixing. They are shown by long-chain triglycerides like grape seed oil, sunflower oil, rape seed, palm oil and corn oil. Normally, ripening inhibitors are used in producing oil-in-water nanoemulsions having oil phases that are highly water soluble like essential oils and flavor (McClements and Jafari, 2018). Due to the hydrophobic action of ripening inhibitors, ripening is impeded. The primary destabilizing mechanism of nanoemulsions is ostwald ripening, means net formation of larger oil droplets from small droplets through the continuous phase. Nanoemulsion quickly becomes unstable because of the growth of droplets and creaming in absence of the ripening inhibitors. When the ripening inhibitor is present in oil droplets it slows droplet growth due to entropy of the mixing effect. The amount of the ripening inhibitor (triglyceride oil) that remains in smaller droplets increases as the water-soluble oil molecules (essential oils or taste) migrate from smaller to larger droplets. Consequently, the system has concentration gradient favouring the mobility of water soluble oil molecules to small droplets from larger droplets counterbalancing the growth of droplets through ostwald ripening. Examples of ripening inhibitors include corn oil, sesame oil, canola oil and sunflower oil (Majeed et al., 2016).

### Plasticizers

Plasticizers are non-volatile compounds having a low molecular weight, placed into the polymer to lower their brittleness and cohesiveness, improve thermoplastic characteristics, toughness and impart flexibility to the polymer. Plasticizers position themselves amid the polymer chains and depending on the integration of structures plasticizers are mainly classified into two groups: external and internal plasticizers. Either due to copolymerization with the structure of polymer or by reacting with polymer, the inner plasticizers merge with the polymer molecule. External plasticizers are generally utilized because these are cheap liquid plasticizers having a series of formulations from highly flexible to semi-rigid materials based on the quantity of plasticizer. Extensively utilized external plasticizers comprise of esters produced from reaction of acids with alcohol or acid anhydrides. The external plasticizers are utilized as primary and secondary plasticizer. Primary plasticizer is added in large quantities to improve flexibility, elongation and softness of polymer. Depending on the processing temperature, secondary plasticizers with lower compatibility with the polymer can cause the polymer to disintegrate (Chaudhary et al., 2020). Films embedded with plasticizers produce large structures owing to adequate space for polymer molecules movement, which results in decreased glass transition temperature of glass and elastic modulus, which softens the polymers. Normally utilized plasticizers in film systems are polyols (polyethylene glycols, glycerol, sorbitol), monosaccharides, oligosaccharides (fructose, glucose, honey and sucrose), lipids and their derivatives (phospholipids, fatty acids). Sucrose, glycerol, xylitol, sorbitol and ethylene glycol are the food grade plasticizers mainly utilized in preparing edible coatings (Sharma et al., 2019).

### Factors impacting the preparation of nanoemulsions

• Selection of Surfactant should be done carefully to achieve ultralow interfacial tension which is the chief requirement to make nanoemulsion.
• Surfactant concentration must be high enough so that microdroplets are stabilized to manufacture nanoemulsion.
• The surfactant flexibility must be sufficient to support the nanoemulsion formation. Intense share should be enforced rupturing the microscale droplets into nanoscale by generating stress levels reaching laplace pressure of droplets with a pressure of 10 to 100 atm.
• Appropriate amount is necessary to avoid ostwald ripening, the dispersed phase should be highly insoluble in the dispersed medium (Jaiswal et al., 2015).

## Methods for preparation of nanoemulsions

Nanoemulsion preparation depends on energy supplied to the system. To prepare nanoemulsions both low as well as high energy techniques have been developed (Shams and Sahari, 2016). Commonly employed low energy emulsification techniques in cosmetic and pharmaceutical industries include phase inversion temperature (PIT) or phase inversion composition (PIC). Energy input is necessary to make nanoemulsion despite being a spontaneous procedure. The energy requirement for the nanoemulsions production can be calculated by the expression: 1G = 1Aγ-T1S. Where 1A depicts the interfacial area increased, γ denotes surface tension and T1S shows the entropy dispersion (Aswathanarayan and Vittal, 2019). The quantity of energy input essential for the production of nanoemulsions is mainly associated with increasing surface area, resulting in formation of the interfacial tension and new globules. Selection of an appropriate method for nanoemulsion production is dependent on certain compound characteristics such as homogenization of oil phases and surfactants, physicochemical attributes and product operational qualities. Additionally, a two-step technique enables the production of two types of multiple nanoemulsions, especially water-in-oil-in-water (W/O/W) and oil-in-water-in-oil (O/W/O). A critical aspect in the production of nanoemulsions is achieving extremely low interfacial tension (<10 to 3 mN/m) at the (O/W) interface, requiring an appropriate usage of surfactants. As surfactants, they aid in the stabilization of droplets with low interfacial tension. Another vital factor for stimulating nanoemulsion formation at the interface is fluidity. The preparation of nanoemulsions can be split into two major categories: low energy procedures and high energy approaches based on the process by which energy is delivered to the system to be emulsified. Nanoemulsions preparation can be done by changing the energy input from the chemical components and the temperature or composition of the (O/W) system.

### Low energy method

Low energy (LE) techniques for the production of nanoemulsions were evolved much later than high energy methods. LE methods depend on the spontaneous production of tiny oil drops in oil–water-emulsifier blends when either their environmental circumstances or the composition is changed. Low-energy methods are characterized by changes in mixture composition and environmental conditions, which influence the development of nanodroplets within the mixed systems containing oil, water and surfactants. LE method is broadly categorized into isothermal such as emulsion phase inversion, spontaneous emulsification and thermal method. Interest in the production and application of LE approaches has accelerated tremendously due to the low cost, ease of implementation, non-destructive and energy-saving (Komaiko and McClements, 2014, Gulotta et al., 2014). Physicochemical factors including temperature, solubility, and composition affect the formation of nanoemulsions using LE techniques (Salvia-Trujillo et al., 2017). Mainly two methods are reported for the development of nanoemulsions by the LE method viz., phase inversion and spontaneous emulsification (Mehrnia et al., 2016).

#### Spontaneous emulsion (SE)

It is also known as solvent diffusion emulsification (ESD) or self-emulsification method, takes place through various mechanisms (Espitia et al., 2019). Predominately it is a process driven by diffusion that occurs when two immiscible liquids are mixed under non-equilibrium condition owing to chemical potential gradient among the phases (Solans et al., 2016). This method is regulated by both altering the temperature without changing the composition or keeping the temperature constant and varying interfacial properties and compositions. This method does not require any special equipment and nanoemulsions by this approach could be developed at ambient condition. The main drawback of the current method is the existence of solvent and the little amount of oil phase (Maali and Mosavian 2013). In this approach, an organic phase such as oil and surfactant are combined with an aqueous phase made up of co-surfactant and water to transfer water-miscible ingredients such as surfactant, solvent, and co-surfactant from an organic phase to an aqueous phase. The transfer of water miscible elements into the aqueous phase increases the oil–water interfacial area because of the extreme instability at the interface between the two phases. When the bi-continuous microemulsion phase breaks down, it results into the spontaneous formation of fine oil drops. Solvents can be used to speed up this process whether surfactants are present or absent; referred to as the ouzo effect (Komaiko and McClements, 2016). The spontaneous emulsification approach can result in nano or microemulsions, regardless of the kinetics. Even though the utilization of this procedure at an industrial level is still in infancy, it has proved as a potentially economical approach, leading to small droplets sized 10 nm (Chen et al., 2016).

#### Emulsion phase inversion (*EPI*)

**Fig. 1:**
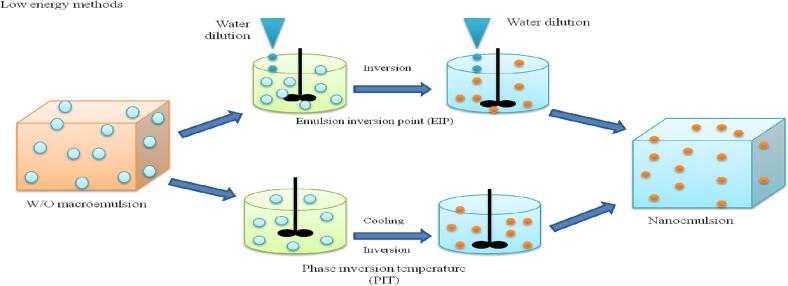
Schematic diagram of nanoemulsion formation using the oil phase and aqueous phase through a low-energy approach.

It means the transformation of O/W emulsions to W/O emulsion or vice versa or a dynamic and desirable phenomenon based on phase transition during the process of emulsification (Jasmina et al., 2017). These phase transitions are caused by the surfactant’s spontaneous curvature and can be induced by altering the spontaneous curvature of non-ionic surfactants with the temperature and by varying system composition. Phase inversion composition, phase inversion temperature, and emulsion phase inversion (*EPI*) technologies are the most popular phase inversion techniques in food industry (Ostertag et al., 2012). Emulsion phase inversion method is induced by several parameters like temperature, salt concentration, oil fraction or water and energy input or by variation in formulation parameters (salinity, temperature, etc.) (Fig. 1).

#### Phase inversion composition (PIC)

Also named as emulsion inversion point, in this technique variation in ingredient makeup alter the emulsifier's hydrophilic-lipophilic behaviour. When salt is added to (O/W) nanoemulsions with an ionic emulsifier, the surfactants' electric charge is altered, resulting in a (W/O) emulsion system (Aswathanarayan and Vittal 2019). Likewise, the water-in-oil emulsions could be converted into oil-in-water by diluting with water. But it is hard to utilize this method for hydrophobic compounds. For using this composition strategy, only a tiny amount of water must be introduced to the oil phase, leading to a gradual rise in the water volume percentage. This process can occur at a specific level, resulting in the formation of a bicontinuous phase that eventually encapsulates oil into water phases, forming O/W nanoemulsions (Perazzo et al., 2015). Since the driving forces are less, this approach requires a longer preparation time than the spontaneous emulsion technique. This technique has been used to create food-grade nanoemulsions that are fortified with vitamin E acetate and have a mean particle diameter of 40 nm. Compared to the microfluidization technique this approach is more successful for producing nanoemulsions with a high concentration of surfactant. Surfactants like casein, whey protein, sucrose monoesters and quillaja saponin are not suitable for the development of nanoemulsions by this approach (Mayer et al., 2013).

#### Phase inversion temperature (PIT)

The temperature where the transition from (O/W) to (W/O) emulsion occurs and uses molecule invasion property and the emulsifiers change their lipophilicity or hydrophilicity as a function of the temperature at a constant composition (Aswathanarayan and Vittal, 2019). This technique produces nanoemulsions with LE input without using the high shear forces. Phase inversion temperature is based on transitional inversion, which can occur in emulsions and is driven by changes in the HLB (hydrophilic-lipophilic balance) system, caused by temperature fluctuations (Maali and Mosavian, 2013). Compared to phase inversion composition this method generally exhibits a low polydispersity index and high emulsification efficiency (Jintapattanakit, 2018). Usually, it is found that when the PIT of the system is 20 to 65 °C nanoemulsions formed are of O/W type and when the temperature range is 10 to 40 °C lower than the storage temperature, the nanoemulsions obtained are W/O type. The stability of nanoemulsions formed by this method is temperature sensitive near the PIT, so this system could be stabilized by the addition of co-surfactants. Surfactants and water are continuously agitated and gradually heated at room temperature to PIT. Rapid cooling of the solution in an ice bath results in the creation of O/W nanoemulsions. However, stable nanoemulsions were exhibited by non-ionic surfactants in the systems’ PIT method. On certain occasions, the phase inversion temperature of the system is adjusted by using inorganic salts (Hasan et al., 2015). Water-in-oil nanoemulsion is produced because oil has higher surfactant solubility (lipophilicity) than the water phase. However, PIT approach completely solubilizes the oil phase in the bicontinuous microemulsion, resulting in O/W nanoemulsion having a very small drop size. The emulsifier's solubility in the water and oil phases practically equals when the curvature of the emulsifier layer reaches zero at a particular temperature. However, at a higher temperature, the surfactants’ layer becomes concave having a negative curvature as a result of drying out of hydrophilic non-ionic surfactant. It is effectively utilized for preparing the nanoemulsion stabilized using ethoxylated surfactants. On the other hand, this method is not applicable while using nonionic and ionic surfactants whose HLB is considerably less sensitive to temperature fluctuations (Koroleva and Yurtov, 2012) (Fig. 1).

### High energy methods

**Fig. 2:**
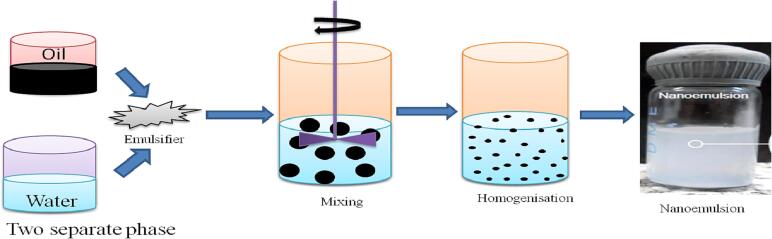
A diagramatic representation of nanoemulsion formation using high-energy approach.

This approach requires the utilization of mechanical gadgets such as ultrasonicator, high pressure homogenizer and micro fluidizer generating forces that are highly disruptive to break up intermingling water and the oil phases, thus developing small droplets (Espitia et al., 2019). The droplet size acquired by the high-energy method is dependent on the intensity of energy. Since, the high energy approach dissipates a substantial quantity of energy as friction losses due to the high shear rates; this energy is transformed to heat, which elevates the emulsion temperature (Fig. 2). Because the mechanical device's high heat interrupts the oil phase, these machines also need an air conditioning system (Wang et al., 2015). Food industries usually make use of these high energy approaches to develop oil in water nanoemulsions.

#### Rotor stator emulsification method (RSE)

The rotor stator emulsification method is the most popular emulsification process widely used by the food industries. Nowadays rotor stator homogenizers are used for nanoemulsion preparation. As compared to other techniques these are having several advantages such as easy installation, low cost investment; viscous system compatibility; large emulsion volume preparation, etc. Rotor stator devices are widely employed to create coarse emulsions prior to further disintegration stages like high pressure homogenization.

This method is the least favourable method for one step production of nanoemulsions. It is highly challenging to reduce the drop size in rotor stator devices below 1 m (Van der Schaaf and Karbstein, 2018). Most frequently used approaches for diminishing the droplet size, change in rotor design, decrease in gap size, increase in rotor speed, increasing droplet residence time in disruption zone. If the formulation is irrelevant, utilising all these characteristics might not be sufficient to create nanoemulsions. For creating droplet breakup effectively, an emulsifier with low interfacial tension and fast absorption kinetics is preferred. An in-line gear-rim mechanism equipped with recirculation loop is utilized for the preparation of emulsion in a batch system. The emulsion constitutes of medium chain triglyceride oil mixed in water having emulsifiers 5% each of Tween 80 and Span 80. By adjusting the rotor's speed and emulsification time, the mean droplet size can be varied between 150 and 270 nm. High-pressure homogenization is used to create an emulsion that has smaller droplet size than other emulsions of the same formulation (Scholz and Keck 2015).

#### High pressure valve homogenization method (HPVHM)

This approach is utilized extensively in industries for nanoemulsions formulation owing to its flexibility and the scaling up process. However, only a few categories of high pressure homogenizers are capable of producing nanoemulsions consisting of nanometer range droplets of ∼ 1 nm range (Salvia-Trujillo et al., 2017). They are also called dynamic high-pressure homogenization (piston homogenizers). Instead of directly creating a nanoemulsion from distinct water and oil phases, these homogenizers effectively reduce the size of a previously utilized coarse emulsion. Hydraulic shear forces and irregular turbulent movements led to the preparation of smooth emulsion particles. It is an exceptionally proficient exothermal method requiring high energy input for the production of nanoemulsion (Borthakur et al., 2016). In this procedure nanoemulsions are created by forcing two liquids as well as cosurfactants and surfactants from a minute orifice in a piston homogenizer at a high pressure between 500 and 5000 psi. Firstly, a considerable volume part of the dispersed phase is produced for an emulsion, which can be diluted subsequently. Coalescence can be lowered by incorporating an excess amount of surfactants. It is an extremely efficient technique that is utilized both at the laboratory as well as industrial level, but it requires a large quantity of energy and during the processing increase in temperature might degrade the constituents (Setya et al., 2014). This approach can only produce (O/W) liquid nanoemulsions with an oil phase lower than 20%. Few problems related to homogenizers include poor productivity, deterioration of components because of the generation of a large amount of heat. In the food industries, High Pressure Valve Homogenizers are utilized as they decrease the size of the droplet in previous coarse emulsion by carrying it from an outlet under the influence of disrupting force like intense turbulence, cavitation and hydraulic shear. This resulted in the design of various nozzles to create the desired size of droplets. In this procedure, a rise in homogenization pressure is a reason for small size droplets (Safaya and Rotliwala 2020). High capacity homogenizers having maximum operating pressure from 350 to 400 MPa are generally called ultra high pressure homogenizers (UHPH) (Wang et al., 2015). Few forms of nanoemulsions are stable at pressures below 150 MPa, where most conventional high pressure homogenizers operate between 50 and 100 MPa (Maali and Mosavian, 2013). By this process, 10% (w/w) of the lipid phase (vitamin E-acetate and orange oil) was homogenised with 90% (w/w) of the aqueous phase to create an oil-in-water nanoemulsion. The aqueous phase constituted of surfactants (Q-naturale or lecithin) and a buffer solution (10 mM sodium phosphate having pH 7.0). Q-naturale consisted of 14 wt% active saponins (the residue primarily being water) (Ozturk et al., 2014).

#### High-Pressure Microfluidic homogenization method (HPMH)

Microfluidic devices have been successfully implemented in the production of nanoemulsion (Salvia-Trujillo et al., 2017). This technique involves passing a coarse emulsion matrix through a small hole under high pressure to cause droplets to break apart. Ultrasonicators are being extensively utilized in research laboratories. They produce pressure waves from electrical waves causing a decrease in size of droplets by extending the duration of sonication, level of power and emulsifier concentration. The inlet chamber of this device is designed in such a way that when coarse emulsion flows from channel streams, it separates into two. Subsequently, in the interaction chamber the streams are prepared to superimpose on one another at a higher velocity creating an energetic disruptive force splitting the droplets and producing a very fine emulsion (Singh and Pulikkal, 2022)**.** The general concept of emulsification by high pressure valve homogenizer and microfluidization is similar, except that microfluidization process utilizes unique microchannels with dimensions ranging from 50 to 300 µm to generate droplets. Numerous designs of microfluidization channels are present to produce nanoemulsions and commonly used are Y-shaped channels. Even though microfluidizers permit one to form approximately monodisperse droplets, these have few disadvantages. Their cost of manufacturing is expensive in comparison to the other high energy equipment. Microfluidization techniques causes’ enlarged size of droplets because of coalescence caused by the large time taken in process of emulsification and causes increases in temperature by utilization of high pressure of nanoemulsions. However in comparison to different homogenization methods, the disruption of the droplet by microfluidization procedure is higher and as result, fine droplets having uniform size are formed (Aswathanarayan and Vittal, 2019). Formation of oil-in-water nanoemulsions having high concentration of internal phase in a microfluidizers has been reported. The emulsions were made from sodium dodecyl sulfate, polydimethylsiloxane and water. Firstly, coarse direct emulsion having 10 mm as an average droplet diameter was prepared by high-shear stirring. After that, it progressed through microfluidizer.

#### Ultrasonic homogenization method (USH)

**Fig. 3:**
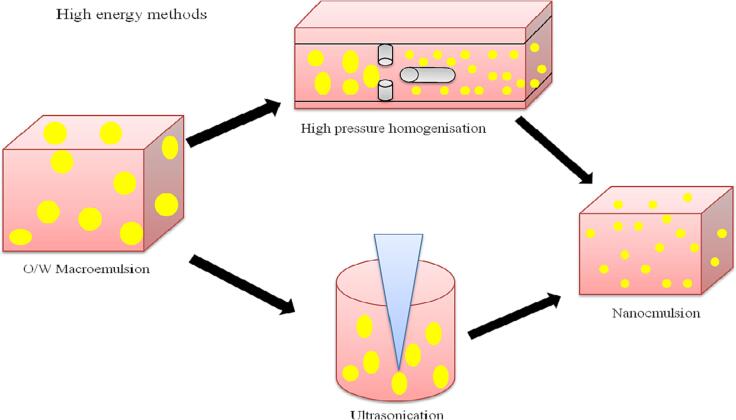
Schematic diagram of nanoemulsion formation using high pressure homogenisation and ultrasonication.

Sonication or ultrasonic method of homogenization can be utilized for the production of kinetically stable nanoemulsions (Jasmina et al., 2017) that are being extensively utilized in research laboratories. They produce pressure waves from electrical waves reducing the droplet size by extending sonication period, level of power and concentration of emulsifiers (Fig. 3). The ultra sonication technique depends on sound waves of high frequency from 20 kHz and above.

The ultrasonic probe utilizes inertial cavitation to offer essential energy for splitting the drops. Sound waves are produced with the help of piezotransmitter, producing mechanical vibrations from electric voltage. The amplification of vibrations is directed by utilizing a probe kept in contact with dispersion. The ultrasonic waves of sound generate sinusoidal fluctuations of pressure in the fluid. When the pressure changes, cavitation bubbles are created, and the bubble goes through many expansions and contractions. Emulsion droplets split off into smaller droplets as these bubbles burst, creating a high shear environment (Shamsara et al., 2015). The piezoelectric probe is capable of being utilized for batch mode ultrasonication by placing it in a batch reactor or beaker and for the continuous process by pumping a stream of fluid through one or many piezotransmitting probes. Liquid jet generators are an alternate technique for continuously producing variations in ultrasonic pressure in which a coarse emulsion is forced from an aperture to strike on a sharp-edged blade. The flow causes this blade to vibrate rapidly, producing an ultrasonic field and cavitation accountable for splitting the droplets. The mechanical ultrasound vibrations can be produced either electrically or mechanically and result in an acoustic cavitation effect that in turn causes the development and disintegration of microbubbles close to the sonication probe, consequently creating turbulence, high shear force and hotspots. Eventually, this method causes effective disruption of droplets in the nanoscale (Wang et al., 2015). Variables like concentration of gas dissolved, hydrostatic pressure, apparatus configuration and temperature also have a significant role in the processes of nano emulsification carried out by utilizing USH devices. The ultrasonication technique uses the least energy compared to other high energy techniques. For large scale applications, commercial homogenizers have been developed based on sonication; wherein the nanoemulsion is passed through a special column having the ability to produce ultrasonic waves (Singh et al., 2017). The morphology of nanoemulsion is affected by frequency, power or amplitude of ultrasound waves and the time of treatment. The contamination brought on by the probe is a significant downside of this method. O/W nanoemulsion prepared under sonication in which the aqueous phase of emulsions comprised of aqueous solution of serum protein and maltodextrin whereas the organic phase was produced by d-limonene, the lowest average droplet diameter of the internal phase was 243 nm (Koroleva and Yurtov, 2012).

### Incorporation into the material

In the last several years, ingredient incorporation in food mixtures has attained importance because of the production of novel delivery systems which are capable of protecting, releasing and encapsulating the active compounds in a better manner (Abinash et al., 2021). Continuous increase in health issues and busy way of life of consumers has led to a change in food consumption pattern, raising the demand for fresh food stuff at a more rapid pace recorded than ever before. Additives including antibrowning/antioxidants, antimicrobial agents, volatile precursors, firming agents, preservatives, colors, nutraceuticals, flavors, etc. are being utilized to enhance the nutritional, visual, rheological and organoleptic properties of nanoemulsion based coatings.

## Antimicrobial nanoemulsions

Refusal of benzoic acid, synthetic additives, sulfite, or their derivative salts generally utilized for controlling the microorganism infestation in foodstuff by consumers, natural agents having antimicrobial properties used for the preservation of foods has gained more demand. At present, biological preservatives obtained from nature are considered safe. Natural biological preservatives are mostly obtained from consumable spices and essential oils and are being detected as efficient preservatives against food borne bacteria and pathogens (Ju et al., 2018). But due to low water solubilization, essential oil needs to be supplied through relevant vehicle to advance their effectiveness. Nanoemulsions besides being transporters of antimicrobial substances enhance the performance of antimicrobial substances as well. Nanoemulsions have emerged as effective encapsulating and delivery systems for antimicrobial agents in food due to their ability to extend the surface area of activity, improve adsorption of antimicrobials, enhance solubility, offer better control over release, allow for manipulation and modification of various factors during preparation, and possess industrialization potential (Jamali et al., 2021). Nanoemulsions have appeared useful particularly against cells of bacteria as they efficiently pass through their external membranes, due to their tiny size and hydrophilic groups present in emulsifying molecules. Due to this reason, no major difference is observed while comparing the antimicrobial effect of various essential oil-based nanoemulsions between Gram (-) and Gram (+) bacteria (Wu et al., 2014). The anti-microbial efficiency of essential oil based nanoemulsion depends on the particular essential oil component utilized, the strain of microbes, the droplet size and constituents of nanoemulsion (Donsì and Ferrari, 2016).

## Antibrowning/antioxidants nanoemulsion

The key quality parameters of fresh-cut fruits and vegetables are their color and appearance. The oxidation phenomenon caused by polyphenol oxidase enzyme is the primary reason for the undesirable changes in the appearance of cut fruits and vegetables. When exposed to oxygen, this enzyme converts phenolic compounds into dark color pigments. To combat this, immobilizing nanodroplets on the surface of fresh foods using nanoemulsion has emerged as a promising approach. The use of nanoemulsion coatings has proven to be an effective system for encapsulating natural antioxidants or antibrowning compounds, such as carotenoids and alpha-tocopherol, in the oil phase. This method has been shown to reduce the browning index of fresh-cut fruits and vegetables compared to the application of antioxidants alone. The conventional approach for controlling the undesirable color changes in cut fruits and vegetables is to immerse them directly in an aqueous solution of antioxidant or antibrowning agents. Ascorbic acid is the most commonly used antioxidant to control the enzymatic browning of cut fruits and vegetables (Hasan et al., 2020). Antioxidants tend to offer stability against oxidation to the formulation either by acting as: synergists like tartaric acid, ascorbic acid, citric acid, citranoic acid and phosphoric acid or blocking agents like tocopherols, butyl hydroxytoluene and ascorbic acid esters or reducing agents like sodium formaldehyde, ascorbic acid, metabisulfite, sodium bisulfite and thiourea (Singh et al., 2017)**.** These are generally called lipophilic antioxidants and are utilized generally in O/W nanoemulsion to decrease the deterioration due to oxidation of susceptible nutraceuticals which helps in prolonging the storage life of foods based on nanoemulsions. Chemical degeneration of sensitive nutraceuticals mostly carotenoids and PUFAs (polyunsaturated fatty acids) can be delayed using lipophilic antioxidants (McClements, 2017). Furthermore, fat soluble antioxidants have been used in foodstuff due to health benefits and to make food commodities rich in antioxidants. Some of the potential lipophilic antioxidants utilized in industries include gallic acid, ascorbyl palmitate, *tert*-butyl hydroquinone, rosemary extracts, butylated hydroxy anisole, butylated hydroxy toluene and alpha tocopherols (Budilarto and Kamal-Eldin 2015). Recent studies confirm that they are highly active fat soluble antioxidants and a single molecule of this can scavenge two free radicals before its inactivation. In the case of oxidation reaction the lag phase can be improved by the usage of these antioxidants. The utilization of antioxidants in nanoemulsions is not explored to a large extent and is still in its infancy (Dasgupta and Ranjan, 2018).

## Texture enhancer agents

Decline in customer demand for fresh-cut fruits is associated with cell wall integrity and loss of texture at the time of storage because of enzymatic degradation. Edible coatings based on nanoemulsion have shown better results in enhancing the texture of fresh-cut vegetables and fruits. Xanthan gum (a texture enhancer based on carbohydrate), was used to prepare nanoemulsions. The result proved that xanthan gum based nanoemulsions are useful in retarding the loss of firmness in fruits (Zambrano-Zaragoza et al., 2014, Hasan et al., 2020). García-Betanzos et al. (2017) demonstrated that the activity of pectin methylesterase and polyphenol oxidase decreased in fresh-cut apples and guava coated with nanoemulsion containing xanthan gum, alpha-tocopherol, and nopal mucilage extracts. The activity of these enzymes is known to contribute to the softening of fruits.

## Flavor enhancement

Flavors are components in food responsible for the distinctive taste and odour. Due to the reason of instability of structures in most flavors, encapsulation appears to be a promising way to protect the characteristic features of these compounds. Encapsulation of flavor in nanoemulsion systems helps in rapid dissociation with increased surface area, making it highly reactive, gravitational separation and better physical stability during aggregation (Tian et al., 2017).

## Methods of application

**Fig. 4:**
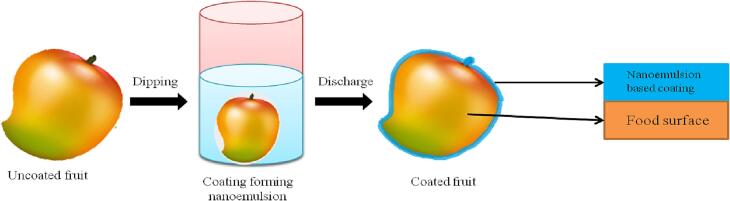
A diagrammatic representation illustrating the dipping method for applying uniform coating.

Different methods have been formulated for applying coatings on the surface of foods. Several commonly used techniques like spraying, dipping and spreading are well known and are being adopted since ages. The spraying is popular in which the size of globule droplets is set around 20 µm making the solution very less viscous and applied at a high pressure (60-80psi) on surface of the commodities. In spraying method substance is twisted up-side down for the even application of coatings onto the whole products’ surface (Ju et al., 2019). The uniformity of coating is dependent on the technique of application, period of drying and temperature (Chaudhary et al., 2020). Spreading contrary to spraying is generally utilized for coating solutions having high viscosity. This procedure is convenient and simple but it has some disadvantages also like uneven coating production. Another technique for applying coating on food products is dipping (Fig. 4) where the food products are wholly drenched in the solution normally for 10–30 s, after drying at ambient temperature it leaves a uniform coating layer over the product’s surface. Dipping must be monitored carefully as it results in the production of thick coating over the products’ surface which may in turn cause anaerobic atmosphere (Andrade et al., 2012).

In case of panning technique, a ‘pan’ is involved that can be a vessel or a pot where coating mixture is applied all over the products. The solution moves continuously for uniform and complete application of the coating onto the products surface. This coating application technique is utilized for both pharmaceutical and food commodities. Subsequently panning drying is done by applying forced air or temperature is increased. The procedure is mostly employed to give finishing to confectionery items. Recently developed coating application technique is the fluidized bed coating that is not having restricted application in food industries only but is being accepted by pharmaceutical industry to polish tablets also (Chaudhary et al., 2020).

## Disadvantages of nanoemulsions:

1. Nanoemulsions are thermodynamically erratic systems susceptible to disintegration due to a number of chemical or physical processes.
2. Nanoemulsions generally enhance their stability to gravitational separation and aggregation while decreasing the size of the droplets; however it may diminish their chemical stability as a result of substantial contact area between the water and oil phase.
3. Due to their vast surface area, nanoemulsions require more emulsifier than normal emulsions, which has an impact on their cost, flavor and toxicity.
4. Several emulsifiers utilized in formulating nanoemulsions, predominantly synthetic surfactants, might result in off-flavor or toxicity of final food product (Das et al., 2020).

## Potential application of nanoemulsions

In the last few years, nanotechnology has become a multimillionaire industry due to business on large scale. It is clear through numerous analyses, reports and applications of patents, that nanotechnology has already gained a great impact on various food aspects and related industries (Neethirajan and Jayas 2011). Edible coatings and films are regarded as convenient, safe and cost effective methods for storage life extension and for maintenance of fruit quality (Yousuf et al., 2018). The large surface area and small size of nanoemulsions offers many physiological properties and physicochemical characteristics which make them appropriate for various applications (McClements and Jafari, 2018). High loss of fresh vegetables and fruits takes place at the time of storage of products which is largely due to shrinkage, moisture loss, biochemical and microbial damage, the process of senescence and mechanical injuries. Edible nanocoatings based nanoemulsions incorporated with colour and flavoring ingredients, enzymes, antioxidants, antibrowning and antimicrobials agents can be utilized for coating food like dairy products including cheese, meat, fresh-cut vegetables and fruits and confectionaries to enhance their storage. Nanoemulsion based coatings can inhibit gaseous exchange; reduce moisture losses and oxidation of food stuff (Donsì, 2018). The advantage of oil-in-water nanoemulsions for various applications is that they get digested quickly inside the gastrointestinal tract due to their increased O/W surface area (Jafari and McClements, 2017). The coloring and flavoring agents used in foods have esters, aldehydes and ketones as functional groups making them prone to photolytic and oxidative degradation. Encapsulating ingredients within nanoemulsion can inhibit the harmful effects and prolong its storage time (Goindi et al., 2016). Plant based essential oils like oregano, orange, thyme and clove and their constituents such as carvacrol, thymol, cinnamon, eugenol and limonene have the high antimicrobial property for food borne microbes and pathogens. But due to their hydrophobicity, they have limited application in food commodities. Nanoemulsion based on essential oils may be adapted to beat this issue and act as natural food preservatives and in packaging foods (Alexandre et al., 2016). Jo et al. (2015) reported that cinnamaldehyde based nanoemulsion (d < 200 nm) inhibited bacterial growth in the melon juice (Valencia-Chamorro et al., 2011). Application of edible film and coating comprising active nanoemulsions is an emerging technology for food fabrication having ideal functions and this sort of method has not been explored yet thoroughly on various fresh-cuts like pineapple, strawberry etc. Some applications of nanoemulsion in fruits and vegetables are given in Table 2.

**Table 2:** Applications of Nanoemulsions as coatings in different fruits and vegetable.

**Fruit/vegetable**	**Nanoemulsions**	**Coating functionality**	**Key finding**	**Reference**
Apple	Fenugreek and Flax seed (1.5 g &1.0 g) + Essential oils (3 mL/L) + tween 20 (0.75 mL/L)	Antimicrobial and antioxidant	Delayed rate of respiration, inhibited production of ethylene and retained quality of fresh fruits at storage period.	Rashid et al., 2020
Fresh cut apple	Nopal mucilage (10 g/L) + Span 80 (0.8 g/L) + α-tocopherol 2 g/L + Tween 80 (4.2 g/L)	Antioxidant and antibrowning	Maintained texture and controlled the browning of fruit	Zambrano-Zaragoza et al., 2014
Fuji Apple	Carnauba-shellac wax + lemongrass oil (3 mL/L) + tween 20 (0.75 mL/L)	Antimicrobial	Decreased microbial population reduced the quality loss, enhanced sensory parameters and prolonged storage time.	Jo et al., 2014
Fresh cut Fuji apple	Sodium alginate(2% w/v) + Lemongrass essential oil (0.1, 0.5 or 1% v/v) + Tween 80 (1% v/v)	Antimicrobial	Nanoemulsion based edible coating with 0.1% (v/v) LEO reduced microbial growth, decreased enzymatic browning and maintained quality in case of the all coating applied.	Salvia-Trujillo et al., 2015
Papaya	Carnauba wax (2.4% & 4.8%) (v/v)	Antimicrobial	Carnauba wax (2.4%) slowed ripening by decreasing weight loss and decay percentage during storage.	Ohashi et al., 2015
Strawberry	Chitosan (3.5 g) + nutmeg seed oil (2.5 mL) + acetic acid (1%) + Tween 80 (5 mL)	Antimicrobial	Suppressed growth of microbes and preserved the quality of fruit during storage for 5 days.	Horison et al.,2019
Plum	Carnauba wax solution (18% w/w) + lemongrass oil(0.5% to 4.0%, w/w) + Tween 80(25% w/w)	Antimicrobial	The edible coatings reduced ethylene production, weight loss, inhibited growth of microbes and enhanced shelf life	Kim et al., 2013
Mango	Thai essential oils(plai + ginger+ fingerroot) + HPMC (5 %w/w)	Antimicrobial	Maintained quality parametrs, slowed ripening and prolonged shelf life upto 18 days.	Klangmuang and Sothornvit 2018
Fresh cut orange	Orange peel essential oil (100%, wt/wt) + Tween 80 + Low methoxyl pectin	Antimicrobial and antioxidant	Higher antifungal and antibacterial activity enhanced shelf life of orange slices without affecting the sensory quality of orange slices.	Radi et al., 2018
Grape berry	lemongrass oil + carnauba wax (18.1 g/100 g) + Tween 80 (25 g)	Antimicrobial and antioxidant	The edible coatings enhanced their glossiness, inhibitted microbial growth and extended shelf life.	Kim et al., 2014
Tomato	Sodium alginate + chitosan + Flourensia cernua Extract + Tween 80 (0.05% w/v)	Antioxidant and antimicrobial	prolonged the storage life of fruit by decreasing weight loss and microbial infestation	de Jesús Salas-Méndez et al., 2019
Melon	Cinnamon oil (5%) + Tween 80 (1.25 mL)	Antimicrobial	Effective natural agent for decreasing microbes in melons.	Paudel et al., 2019
Guava	Chitosan (5 % m/v) + alginate(5 % m/v) + nanoZnO (1%, v/gel)	Antimicrobial and antioxidant	Prolonged storage to 20 days.	Arroyo et al., 2020
Okra	Basil oil (0.5% v/v) + Tween 20 (0.2% v/ v) + sapindus extract (0.2–0.4% w/v)	Antimicrobial	Inhibited microbial growth, improved postharvest parameters and storage period.	Gundewadi et al., 2018
Green beans	Mandarin essential oil (2 wt%) + sunflower oil (2 wt%) + Tween 20 (0.75 wt%) + glycerol monooleate (0.75 wt%) + chitosan (3%)	Antimicrobial	Lowered *Listeria innocua* significantly during the storage time, which resulted in improvement in antimicrobial activity and affected highly the green beans firmness.	Donsì et al., 2015

### Application of nanoemulsion in shelf life of fruits

Nanoemulsions are mostly used to create edible coatings. For evaluating these coatings apple has become one of the models and it was seen that apple manages to considerably decrease cellular oxidative stress, maintaining its physicochemical characters (Zambrano-Zaragoza et al., 2014, Salvia-Trujillo et al., 2017). Recently it has been found that an optimized system of nanoemulsion films and coating based on food-grade materials preserved the long term storage capability of apple fruits. New data suggest that coatings of nanoemulsions consisting of 1.0 g flaxseed polysaccharide and 1.5 g fenugreek showed the least microbial decay, weight loss (0.70%) and maintained the firmness (6.29 kg/ cm^2^), pH (4.1), total soluble solids (11.05%), phenolic contents (335.7 mg/100 g) and antioxidant (44.5%) throughout the storage time in contrast to the other treatment. Biopolymers have effectively maintained the physico-chemical features of apple fruits by inhibiting production of ethylene and delaying the respiration rate (Rashid et al., 2020). In one more research conducted on fresh-cut “Fuji” apples, the essential oil obtained from lemongrass was used in the emulsions and nanoemulsions containing sodium alginate matrix revealed that during refrigerated storage luminosity reduced. Apples coated just with sodium alginate did not show the same results, explained by the data that the phenolic compounds included in essential oils function as substrate for the activity of polyphenol oxidase. Further, it was observed that there is an enhancement in cell membrane permeability caused by volatile compounds present in the essential oils encouraging cell cytoplasm modifications, allowing enzymes and phenols to interact (Yilmaz et al., 2016). A study proposed by Ohashi et al. (2015) for the characterization of an emulsion of carnauba wax was to evaluate the effect of low concentration (2.4 and 4.8%) of this emulsion as a protecting coat for ‘Golden’ papaya at the time of storage. Applying carnauba wax (2.4%) deferred ripening of papaya, by minimizing decay incidence and weight loss at the time of preservation. In contrast to this; application of carnauba wax at concentration 4.8% has not been successful in maintaining the postharvest quality parameters, resulting in poor effects; like high pH values and decay percentage. Results proved that such protective coating can be utilized in conjunction with water, increasing their efficiencies to maintain the postharvest quality of food products. Rocha pear an essential fruit having extended storage potential, can be stored for about 10 to 11 months under regulated atmosphere, however, these may get chilling injury during cold storage for longer period (Saquet and Almeida, 2017). A study based on the impact of coating ‘Rocha’ pear with alginate based nanoemulsion consisting of citral (Cit) or lemongrass essential oil (LG) was examined. The results showed that, control fruit (uncoated) had decreased loss of firmness and high electrolyte leakage in contrast to the coated pears showing their effectiveness on slowing the ripening. Additionally, a coated pear with Lemon grass nanoemulsions had no signs of scald on surface and helped preserve sensory attributes for up to 6 months and 7-days of storage period (Gago et al., 2020). Storage of table grapes displays major problems such as loss of weight, firmness and change of colour. Sánchez-González et al. (2011) examined the biodegradable coating made from chitosan (CH) or hydroxypropylmethylcellulose (HPMC) without and with bergamot essential oil in maintaining quality of fresh fruits and safety in the duration of postharvest cold storage. The results revealed that the coating enhanced the mechanical resistance of the product throughout the storage period. Pineapple possesses rich quantity of ascorbic acid, fibers and minerals, is liked for its unique aroma and taste. Fresh-cut-pineapple considerably has a short storage time since cutting and peeling of fruit enhances its metabolic activities, thereby causing deterioration in fruit quality like texture decay, enzymatic browning, microbial contamination and production of undesirable volatiles. Prakash et al. (2020) analyzed the outcome of sodium alginate based nanoemulsions containing various concentrations of citral (1.0, 0.5 and 0.1 %) on the physicochemical, sensory and microbial activities of fresh-cut pineapples stored at 4 °C and relative humidity 90% for up to 12 days. Throughout storage time of 12 days, fresh cut pineapple sample coated with 0.5 and 1.0% citral exhibited superior colour retention, low respiration rate and decreased microbial infestation suggesting that sodium alginate nanoemulsion edible coatings containing 0.5% citral can be used for prolonging the storage period of fresh-cut pineapple commercially. Strawberries are highly vulnerable to postharvest losses during transportation and storage. They have a high respiration activity, which depends on the temperature conditions, handling, degree of maturity and storage time. Strawberries are valued for their phytochemical compounds, flavor and aroma (Van de Velde et al., 2013).

Horison et al. (2019) examined the effectiveness of chitosan based nanoemulsions enriched by nutmeg seed oil on the quality of the fresh strawberry kept at 10 °C for 5 days. The results depicted that samples coated with nanoemulsion showed a decrease in mould, yeast and microbial growth at the end of storage period in contrast to the control revealing that nanoemulsion assists in quality preservation of strawberries kept for 5 days storage. Another research conducted by (Robledo et al., 2018) on the efficiency of quinoa protein/chitosan nanoemulsion coating containing thymol nanoemulsion on quality, sensorial and safety properties of refrigerated strawberries stored in commercial circumstances. To quinoa protein/chitosan coatings the incorporation of Thy/Ne exhibited antifungal activities in strawberries stored under commercial refrigerated conditions and for almost 10 days. Incorporation of thymol antimicrobial agent in nanoemulsions coating also preserved the sensory characteristics of strawberries, particularly flavour and aroma, until 12th day of cold storage. Additionally, the post-harvest storage period of coated strawberry was prolonged by four days in contrast to control. Kim et al. (2013), formulated carnauba wax nanoemulsions containing lemongrass oil (LO) and investigated the effectiveness of nanoemulsions on the physicochemical storage qualities and microbiological safety of plum fruit kept at 4 °C and 25 °C. The results revealed that a nanoemulsion enriched with LO enhanced the shelf life of coated plums by inhibiting the microbial growth. Mango being perishable climacteric fruit is highly susceptible to several postharvest losses. Mango fruits are highly infested by anthracnose disease (Colletotrichum gloeosporioides) throughout the storage period, transport and marketing. Therefore, any promising method which can successfully control anthracnose infection will play a major role in extension of shelf life of mango fruits. Klangmuang and Sothornvit, (2018) reported that biopolymer like hydroxypropyl methylcellulose (HPMC) do not show any antifungal activities of their own. To improve its antifungal property, Thai essential oils (EOs) were included to HPMC-incorporated nanocomposite coating and examined its effects in mangoes cv. Namdokmai Sithong for anthracnose infection. Results obtained showed that coated mangoes with HPMC nanocomposite coating enriched by Thai oils (Eos) prolonged the storage of fruits for 17 to 18 days kept at 13 °C. Besides this coated fruits also exhibited high overall acceptability score than control. Finger root, ginger and plai-based essential oils embedded in edible nanocomposite coating based on HPMC reduced quality loss due to C. gloeosporioides in comparison to HPMC-based edible nanocomposite coating without essential oils. As a result, edible nanocomposite coating based on active HPMC in combination with EOs can inhibit C. gloeosporioides and prolonged the shelf life of mango without any unfavourable sensory quality. Radi et al. (2018) examined the effect of pectin-based edible coatings consisting of orange peel EO at concentrations 0.5 and 1.0% on quality constraints of the orange pieces preserved for 17 days at 4 °C. The results depicted that pectin based nanoemulsions possessing orange peel essential oils decreased the quality loss and enhanced the sensory attributes of orange slices during storage. Kim et al. (2014) developed nanoemulsions coating containing lemongrass oil (LO) for grape berries (Vitis labruscana) to screen the microbial safety and extend the fruits’ shelf life. Results indicated coatings significantly minimised weight loss, maintained firmness, preserved the phenolic compounds, antioxidant activity as well as concentration of anthocyanin in the berries. Also the LO nanoemulsion coating showed capacity to restrain foodborne pathogen contaminating grape berries and extended the storage life. The findings concluded that the emulsion coating based on LO is an efficient technology for postharvest fruits, viable commercially for ready-to-eat grape berries to enhance food microbial safety and slow down the degradation rate of physicochemical characteristics of the fruit. Guava having high nutritional quality resulting from ascorbic acid, polyphenols and carotenoids, is a climacteric fruit. After harvesting the process of maturation continues, having a high rate of respiration due to metabolic activity responsible for its high perishability, decreased its storage time. Arroyo et al. (2020) observed that the edible coatings consisting of chitosan and alginate displayed as a promising alternative for packing guava fruit. The incorporation of nanozincoxide (nanoZnO) to the coating delayed rotting in guava fruit indicating a significant antimicrobial activity. Coatings having alginate/chitosan/nanoZnO deferred the process of ripening, weight loss and deterioration in contrast to control (uncoated fruit). Additionally, the coatings increased storage up to 20 days in condition favourable for consuming against 7 days for control fruits (uncoated). Paudel et al. (2019) made different cinnamon oil based nanoemulsion formulations through ultrasonication in which Tween 80 emulsifier was used. For Salmonella spp. and Listeria monocytogenes strains minimum inhibitory concentration (MIC) of cinnamon oil nanoemulsions was 0.039% v/v and 0.078% v/v, respectively. In contrast to water control, 0.5% nanoemulsion displayed up to 5.5 and 7.7 log decline in Salmonella spp and Listeria monocytogenes, respectively. The results suggest that nanoemulsions based on cinnamon oil can be employed as a potent natural microbe inhibitor for melon fruits.

### Application of nanoemulsions in shelf life of vegetables

Tomato being a climacteric fruit whose quality is impacted by mass transfer as a result of transpiration, in which the water vapors migrate from the fruits into the surrounding air resulting weight loss of tomatoes. To overcome this problem, many techniques, including nanoemulsions are being applied to prolong the storage period of this horticultural food product (Liplap et al., 2013). Robledo et al. (2018) investigated the effectiveness of quinoa protein/chitosan nanoemulsions containing thymol on growth of mould inoculated in cherry tomato. Results showed a considerable reduction in fungus formation at 5 °C after 7 days and enhanced the storage life of fruit. A new study was carried out by Banerjee et al. (2020) on the effects of edible nanoemulsion coating consisting of sweet orange essential oil and sodium alginate on different quality parameters of tomato. The results revealed that tomatoes coated with edible coatings had a significant increase in firmness, decrease in total mesophilic bacteria and decreased loss of weight by 3 folds lower than uncoated tomatoes. Sensory evaluation disclosed that the usage of edible coatings increased total acceptance score of tomato. Okra has a major contribution to the domestic and overseas vegetable market; however, it is highly perishable due to fungal spoilage and desiccation. Gundewadi et al. (2018) reported that alginate coating incorporated in nano-emulsified basil oil can be used for preserving the postharvest quality and preventing spoilage. Coated okra with alginate coatings having nanoemulsified basil oil maintained texture, enhanced colour as well as retained the overall acceptability substantially with samples without coating. Therefore alginate coatings nanoemulsified by basil oil and sapindus extracts can appear as a potential nonchemical strategy extending the postharvest shelf life of okra. The main problem while consuming fresh-cut red bell-pepper is food-borne pathogenic contamination and loss of quality. Sathiyaseelan et al. (2021) investigated the effect of calcium chloride (CaCl2) and low molecular weight chitosan (LMWCS)/ tea tree oil (TTO) nanoemulsion coatings on the fresh-cut red bell-pepper quality preserved for 21 days at 4 °C. The combination of CaCl2-LMWCS/TTO emulsion application preserved the overall quality, sensory behaviour and texture of fresh cut red bell-pepper for 18 days compared to the control. Zhang et al. (2021) studied the effects of foliar applied antimicrobial nanoemulsion (carvacrol) on spinach. The results revealed that the plants remained healthy while spraying carvacrol nanoemulsions on the spinach leaves at low concentration (0.005 to 0.5%). However, increased in carvacrol concentration (5%) decreased chlorophyll content and biomass of spinach and increased malondialdehyde formation and electrolyte leakage. Donsi et al. (2015) reported that a coating mixture containing modified chitosan enriched with mandarin EO as an antimicrobial agent proved as the most capable method resulting in a consequent decrease in L. innocua during the complete storage time. Thus significantly impacts the firmness of green beans resulting in the creation of a major synergism of antibacterial action.

## Conclusion

Nanoemulsions have been recognized as an effective and most ideal form of technology in agro-food based industries. The edible food grade nanoemulsion application has been proved to enhance the sensorial, texture, nutritive quality and stability of a variety of food stuff. The potential utilization of edible nanoemulsions in vegetables, fruits, nutraceutical, confectionery, beverage, food packaging and dairy industries can meet the bulk demands from the market. One more critical aspect is choosing the suitable techniques and ingredients for preparation and formulation of edible nanoemulsions so that they could possess optimum application based on rheological properties, efficiency, stability and appearance. The usage of edible nanoemulsions in food commodities can be estimated to increase exponentially in the coming year, particularly to impart amplified functionality combined with improved sensorial qualities to food commodities. The production of food grade nanoemulsions utilized for encapsulating and enhancement of the functionality of certain active substances is an evident approach for novel product generation. A viable approach to meet the customer's demand is to use natural texture enhancers, antimicrobial/antioxidants, and antioxidants as alternatives to chemical additives. The findings of current research indicate the potential advantages of utilising nanoemulsion coatings produced using natural components rather than traditional emulsion based edible coating for increasing the safety, quality and storage life of vegetables and fruits.

**Funding:** This study was supported by Scientific Research Projects Department of Ataturk University, Erzurum, Türkiye (Project no: FBA-2019-8241).

**Data Availability Statement:** All the authors declare that if more data is required, then the data will be provided on a request basis.

## Declaration of Competing Interest

The authors declare that they have no known competing financial interests or personal relationships that could have appeared to influence the work reported in this paper.

## Data Availability

No data was used for the research described in the article.
